# Genotoxicity evaluation of Oryeong-san water extract using *in vitro* and *in vivo* tests

**DOI:** 10.1186/s12906-015-0804-3

**Published:** 2015-08-13

**Authors:** Mee-Young Lee, Chang-Sebo Seo, Ji-Young Kim, Hyeun-Kyoo Shin

**Affiliations:** Herbal Medicine Formulation Research Group, Korea Institute of Oriental Medicine, 483 Expo-ro, Yusung-gu Daejeon, 305-811 Republic of Korea; Division of Nonclinical Studies, Korea Institute of Toxicology, P.O. Box 123, 19 Sinseongro, Yuseong-gu Daejeon, 305-343 Republic of Korea

**Keywords:** Oryeong-san, Ames test, Chromosome aberration assay, Micronucleus

## Abstract

**Background:**

Oryeong-san, a mixture of five herbal plants, is a well-known therapy for renal-associated diseases such as those manifesting edema, dysuria, and oliguria.

**Methods:**

In the present study, we investigatee the potential genotoxic effects of a water extract of Oryeong-san (ORSE) in three mutagenicity assays (an *in vitro* bacterial reverse mutation assay (Ames test) with *Salmonella typhimurium* and *Escherichia coli* strains, an *in vitro* mammalian chromosomal aberration test using Chinese hamster lung cells, and an *in vivo* micronucleus test using ICR mice bone marrow).

**Results:**

ORSE showed no genotoxicity in the Ames test up to 5000 μg/plate; the *in vitro* chromosome aberration test showed no significant structural aberrations with and without the S9 mix up to 5000 μg/mL, or the *in vivo* micronucleus test up to 2000 mg/kg body weight.

**Conclusions:**

In conclusion, under the current test conditions, ORSE seems safe for use; however, other genotoxicity tests (e.g. sister-chromatid exchange or Comet) or chronic toxicity tests are warranted.

## Background

The global demand for herbal medicinal products has increased significantly in recent years. “By 2003 in the United States alone, over 1500 herbal products sold were nutraceuticals, which are exempt from extensive preclinical efficacy and toxicity testing by the US Food and Drug Administration” [1]. Obidike and Salawu reported that despite the growing market demand for herbal medicines, there are still concerns associated with not only their use, but also their safety. The primary purpose of toxicological assessment of any herbal medicine is to identify side effects and to determine limits of exposure at which such effects occur [2]. Less than 10% of herbal products in the world market are truely standardized to known major active components and quality control measures are not always diligently adhered to [3]. The traditional herbal medicine Oryeong-san (also known as Wulingsan in traditional Chinese medicine and as Gorei-san in Japanese Kampo medicine) is a mixture of five herbal preparations (*Alisma orientale (Sam) Juzepzuk*, *Poria cocos*, *Atractylodes japonica Koidzumi et Kitagawa*, *Polyporus umbellatus Fries*, and *Cinnamomum cassia J.Presl*). Oryeong-san is well known for the treatment of renal diseases, dysuria, manifesting edema, and oliguria [4]. Oryeong-san also has antihypertensive [5], antidiabetic [6], antioxidative [7], and antigastric [8] properties, and confers hepatic protection.

Genotoxicity is a special type of toxicity, because it is often the most difficult to detect. The aim of a genotoxic test is to detect mutagenic carcinogens as well as to detect germ cell mutagens with the goal of limiting human exposure to these potentially dangerous chemicals [9]. The purpose of the present study was to evaluate the safety of an aqueous extract of Oryeong-san (ORSE) and its potential genotoxicity. We assessed these properties using a standard battery of tests recommended by the Korea Food and Drug Administration: a bacterial reverse mutation test (Ames test), a chromosome aberration test, and an *in vivo* micronucleus (MN) test.

## Methods

### Reagents and materials

The reference standards, cinnamaldehyde and coumarin, were purchased from Wako (Osaka, Japan) and Sigma-Aldrich (St. Louis, CA, USA). The purity of the two reference standards was ≥98.0% by HPLC analysis. The HPLC-grade solvents, acetonitrile and water, methanol, were obtained from J.T. Baker (Phillipsburg, NJ, USA). The Oryeong-san samples used in this study consisted of five herbal medicines (Table 1) and were purchased from Kwangmyungdang Medicinal herbs (Ulsan, Korea). All herbal medicines were taxonomically confirmed by Professor Je-Hyun Lee, Dongguk University, Korea. Voucher specimens (2013-KE17-1 through KE17-5) have been deposited at the Herbal Medicine Formulation Research Group, Korea Institute of Oriental Medicine.

**Table 1:** The combination of crude components of ORSE

Latin name	Amount (g)	Herbal name	Source
*Alisma orientale Juzepzuk*	9.375	Alismatis Rhizoma	Yeongcheom, Korea
*Poria cocos*	5.625	Poria Sclerotium	Pyeongchang, Korea
*Atractylodes japonica* Koidzumi	5.625	Atractylodis Rhizoma Alba	China
*Polyporus umbellatus* Fries	5.625	Polyporus	China
*Cinnamomum cassia* Presl	1.875	Cinnamomi Cortex	Vietnam
Total	28.1		

### Preparation of standard and sample solutions

Standard stock solutions of coumarin and cinnamaldehyde were dissolved in methanol at 1.0 mg/mL and stored below 4°C. A decoction of Oryeong-san, which was composed of 5 herbal medicines (Table 1), was prepared in SungIl Bioex Co. Ltd. (Hwaseong, Korea). Briefly, the mixture (120.0 kg. i.e. about 4270.46 times of composition of single dose) of 5 herbal medicine was extracted in distilled water (1200 L) at 80°C for 2 h by reflux. The solution was freeze-dried to about 21.8 kg of water extract powder (yield: 18.17%). Lyophilized Oryeong-san extract (200 mg) was dissolved in distilled water (20 mL). The solution was filtered through a 0.2 μm membrane filter (Woongki Science, Seoul, Korea).

### HPLC analysis of ORSE

HPLC simultaneous determination was performed using an LC-20A HPLC system (Shimadzu, Kyoto, Japan), consisting of an LC-20AT pump, DGU-20A_3_ online degasser, SPD-M20A detector, SIL-20AC autosampler, and CTO-20A column oven. The data processor employed LC solution software (version 1.24, Shimadzu). The separation of three compounds was conducted using a Gemini C_18_ (250 mm × 4.6 mm; 5 μm, Phenomenex, Torrance, CA, USA) and column oven temperature was maintained at 40°C. The mobile phases comprised water (A) and acetonitrile (B). The gradient condition was as follows: 0–30 min, 25–100% B; 30–35 min, 100–25% B; 35–50 min, 25% B. The analysis was conducted at a flow rate of 1.0 mL/min with PDA detection at 280 nm. The injection volume was 10 μL.

### Bacterial reverse mutation assay (Ames test)

Bacterial reverse mutation assays were conducted as described previously [10]. *Salmonella typhimurium* strains TA98 and TA1537 (to detect frame-shift mutagens), TA100, and TA1535, and *Escherichia coli* strain WP2uvrA (to detect base pair-substitution mutagens) were obtained from Molecular Toxicology Inc. (Boone, NC, USA) and were used as the tester strains. To evaluate the toxicity and solubility (precipitation) of ORSE, a pilot experiment was performed with all bacterial strains (data not shown). The positive control factors were 2-nitrofluorene (2-NF), 2-aminoanthracene (2-AA), 9-aminoacridine (9-AA), 4-nitroquinoline X-oxide (4NQO), and benzo (a) pyrene (BP). A dose range-finding test was performed to determine the highest concentration for the present study, which was performed with the five tester strains at concentrations of 0, 1250, 2500, and 5,000 μg/plate with and without the S9 mixture. The number of revertant colonies did not increase to more than twice the value observed in the controls for any of the tester stains. However, there were increased numbers of revertant colonies of TA1535 with the S9 mixture. Based on these results, a dose of 5,000 μg/plate was selected as the maximum dose. Briefly, various concentrations of ORSE were incubated with the tester strains at 37°C for 48 h in the presence or absence of metabolic activation by the S9 mixture along with vehicle and positive controls containing the following combinations of substances and doses: 2-AA at 2 μg/plate vs. TA1535 with or without the S9 mixture and at 4 μg/plate vs. WP2uvrA with the S9 mixture; 9-AA at 50 μg/plate vs. TA1537 without the S9 mixture; BP at 2 μg/plate vs. TA98 with or without the S9 mixture and vs. TA100 and TA1537 with the S9 mixture); 2-NF at 2 μg/plate vs. TA98 without the S9 mixture; 4NQO at 0.5 μg/plate vs. WP2uvrA without the S9 mixture; and sodium azide at 0.5 μg/plate vs. TA100 and TA1535 without the S9 mixture. Each concentration of ORSE was tested in triplicate. A result was deemed positive if there was a concentration-related increase over the range tested and/or a reproducible increase at one or more concentrations in the number of revertant colonies per plate in at least one strain with or without the S9 mixture. An antibacterial effect (cytotoxicity) was defined as a clearing or diminution of the background lawn, the appearance of microcolonies, and/or a decrease of > 50% in the number of colonies compared with the relevant vehicle control.

### Chromosome aberration test

Chromosome aberration tests were conducted as described previously [10] with minor modification as described by Ishidate et al. [11] and Dean and Danford [12]. Chinese hamster lung (CHL) cells were obtained from American Type Culture Collection (Manassas, VA, USA) in 2004. The cells were thawed in culture medium and then grown for more than 7 days as a monolayer. Cells were cultured in reconstituted MEM (Gibco-Invitrogen, USA) supplemented with 2.2 g of sodium bicarbonate, 292 mg of l-glutamine, streptomycin sulfate (100 μg/mL), penicillin G · Na (10^5^ units), and 10% (v/v) fetal bovine serum (FBS; Gibco-Invitrogen, USA) per liter. The cultures were incubated at 37°C in a humidified atmosphere with 1.5% CO_2_. A preliminary dose range-finding study was performed to determine the highest concentration for this study. Using the results from the dose range-finding study, the dose range for the present study was designed to quantify the solubility and cytotoxicity of GBT. Ethyl methanesulfonate (EMS) was used as a positive control substance without metabolic activation and cyclophosphamide (CPA) with metabolic activation. Cells were trypsinized and counted, and the relative cell count (RCC) was calculated. The cells were centrifuged at ~1000 rpm for 5 min and resuspended in 5 mL of 75 mM KCl solution. After 10 min at room temperature, 5 mL of fixative (methanol:glacial acetic acid = 3:1 v/v) was added to the cell suspension, and the suspension was refrigerated for ~20 min. The fixative was changed twice by centrifugation at ~1500 rpm for 5 min. Two slides were prepared from each fixed-cell suspension. The slides were air-dried, stained with 3% Giemsa solution, washed in tap water and distilled water, dried, and mounted in DPX (Fluka) for chromosome aberration scoring. Chromosome aberrations were identified morphologically according to the principles described in the ‘Atlas of chromosome aberration by chemicals’ (JEMS-MMS, 1988). Cells with more than four of the same type of aberration were scored as multiple aberrations. Any metaphase with one or more aberrations, regardless of the type, was classified as an aberration metaphase. Slides were scanned systemically, and each set of metaphases was examined at 1000× magnification. Structural chromosome aberrations were evaluated in 100 well-spread metaphases, each containing 23–27 chromosomes. The microscopic stage coordinates and each type and number of aberration were recorded for each aberrant metaphase. The results are expressed as the number of findings per 100 metaphases. Regardless of the presence of aberrations, an additional 100 metaphases were examined to determine the frequency of diploidy (DP), polyploidy (PP, > 37 chromosomes), and endoreduplication (ER).

### *In vivo* MN test

MN tests using mice was conducted as described previously [10]. Specific pathogen-free male CrljOri:CD1 (ICR) mice weighing 27.2–30.0 g were obtained from Orient Bio Co., Ltd. (Seongnam, Korea) at 6 weeks of age. The preliminary study showed that oral administration of ORSE at a dose of 2000 mg/kg did not induce any toxic effect (data not shown). The highest dose was determined based on the dose range-finding study, and 2000 mg/kg, which was the limit dose for treatment up to 14 days according to the OECD guidelines, was selected as the maximum dose. Mice were used in experiments after 1 week of quarantine and acclimatization. Two slides of the cell suspension from each animal were made. Small round or oval bodies, measuring about 1/5 to 1/20 the diameter of a polychromatic erythrocyte (PCE), were counted as micronuclei. The same observer scored a total of 2000 PCEs per animal to determine the frequency of micronucleated polychromatic erythrocytes (MNPCEs). The ratio of PCEs to normochromatic erythrocytes (NCE) [PCE/(PCE + NCE) ratio] was calculated by counting 500 cells. The mortality and external appearance of animals were checked and recorded once a day during the study period, and these observations were made three times after the final administration on the final dosing day. Body weight was measured on the days of reception, grouping, dosing, and autopsy. This study was approved by the Korea Institute of Toxicology (Daejeon, Republic of Korea), and was conducted according to the guidelines of the Institutional Animal Care and Use Committee in the Korea Institute of Toxicology, which is accredited by AAALAC International (1998) under the GLP Regulations for Nonclinical Laboratory Studies.

### Statistical analyses

The statistical analyses used for the study were selected based on the methods used in published reports [13] using SAS software (version 9.1.3, SAS Institute Inc., Cary, NC, USA). Each metaphase was classified as a normal metaphase or aberrant metaphase with one or more aberrations, and the frequency of aberrant metaphase was analyzed statistically. The numerical aberrations were classified into DP, PP, and ER, and the frequencies of PP + ER were analyzed. The χ^2^ test and Fisher’s exact test were performed to compare the vehicle control and ORSE-treated groups [14]. Fisher’s exact test was used to compare the vehicle and positive control groups. Differences were regarded as significant at *P* < 0.05The *in vivo* MN results were evaluated as described previously [10] using the method of Lovell et al. [15] with minor modification. Data with heterogeneous variances were analyzed using Kruskal–Wallis analysis of variance followed by multiple comparisons using Dunnett’s test [16] The significance was accepted when all of the PCE/(PCE + NCE) ratios were > 0.1. The result was judged as positive when there was a significant and dose-related increase or a reproducible increase in the frequency of MNPCEs or aberrant metaphases at one or more dose levels. Differences were regarded as significant at *P* < 0.05.

## Results

### HPLC analysis of ORSE

**Fig. 1:**
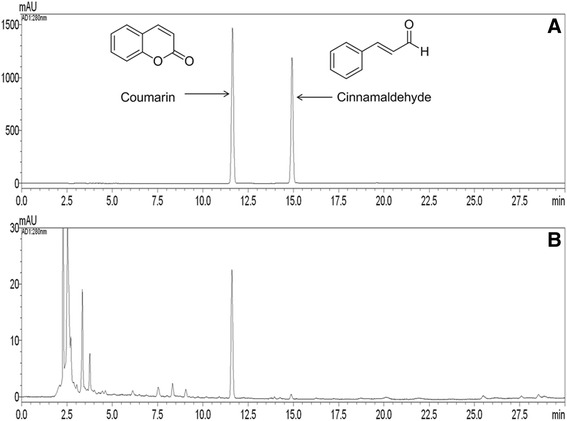
HPLC chromatograms of (**a**) the reference standard mixture and (**b**) ORSE: 230 nm (I), 254 nm (II), and 280 nm

Using established conditions, three components were eluted within 35 min in a sample analysis using mobile phases consisting of solvent A (water) and solvent B (acetonitrile). A typical HPLC chromatogram for ORSE is shown in Fig. 1. The retention times of the two components were 11.60 min (coumarin) and 14.88 min (cinnamaldehyde). The regression equations for coumarin and cinnamaldehyde were *y* = 42536.98*x* – 4343.49 and *y* = 107074.20*x* – 46100.12. The correlation coefficients (*r*^2^) of the calibration curves for the two constituents were 1.0000 and 0.9999. These results showed that the calibration curves showed good linearity. The contents of two components identified in ORSE were 0.37 mg/g and 0.05 mg/g (Table 2).

**Table 2:** Contents of two components in the ORSE by HPLC (*n* = 3)

Compound	Mean (mg/g)	SD	RSD (%)	Source
Coumarin	0.37	0.01	1.80	Cinnamomi Cortex
Cinnamaldehyde	0.05	0.00	0.06	Cinnamomi Cortex

### Bacterial reverse mutation assay (Ames test)

**Fig. 2:**
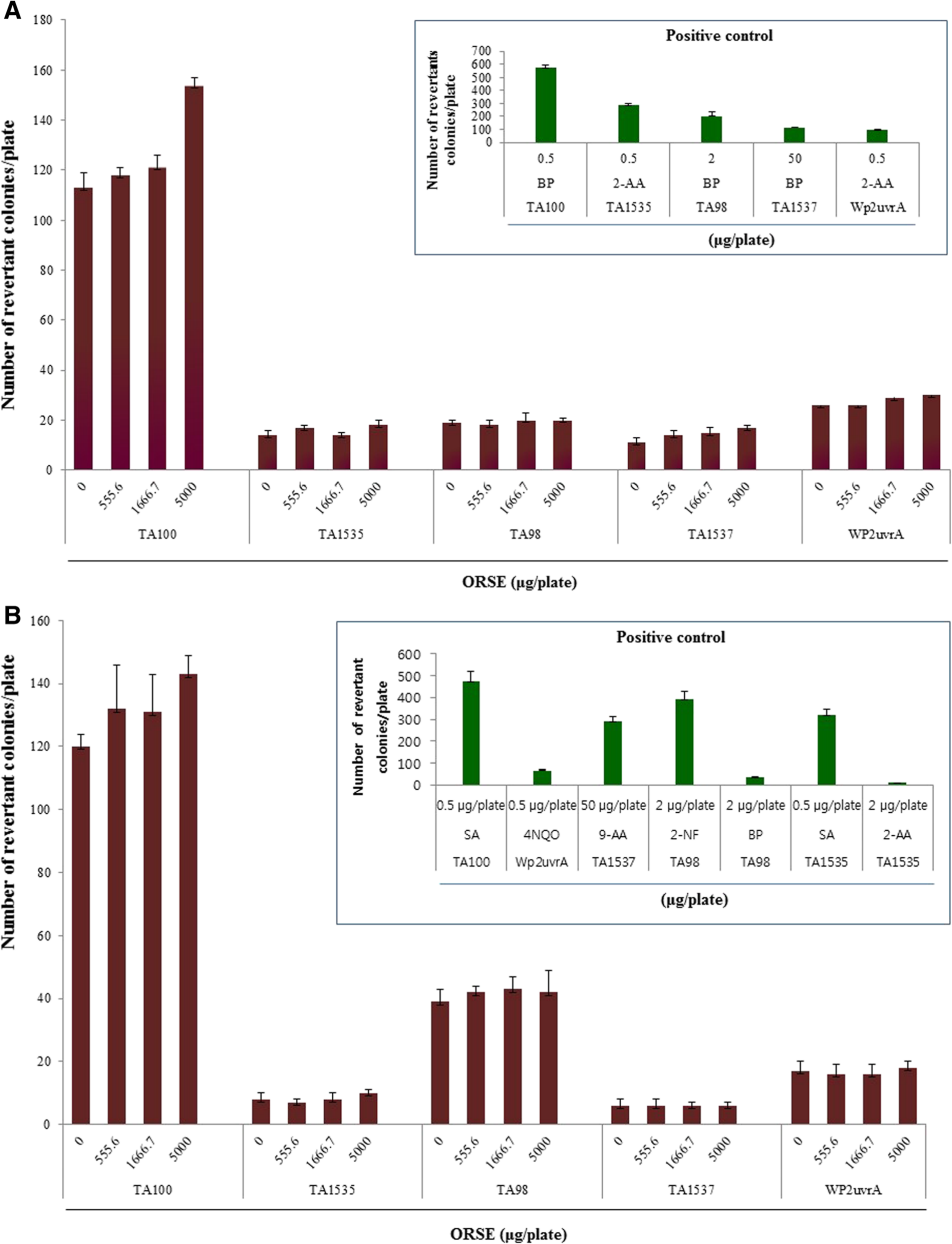
Effect of ORSE in the bacterial reverse mutation assay (Ames test) (**a**) with (+S9 mix) and (**b**) without (−S9 mix) metabolic activation

No positive mutagenic response was observed in any of the *S. typhimurium* or *E. coli* strains tested compared with concurrent vehicle control groups regardless of the presence (Fig. 2a) or absence (Fig. 2b) of the S9 mix up to 5000 μg/plate.

### Chromosome aberration tests

According to our preliminary study (data not shown), ORSE neither inhibited cell growth nor killed Chinese hamster lung (CHL) cells. We examined the concentration range of 1250, 2500, and 5000 μg/mL, which was most compatible with a good cell-proliferating ability and which produced a sufficient number of metaphases for the confirmatory assay. Therefore, we used 5000 μg/mL as the highest exposure level and serial dilutions for further dose–response tests.

There were no statistically significant increase in the number of metaphase cells with structural aberrations at 6 h or 22 h with or without the S9 mix in the ORSE-treated group up 5000 μg/mL (Table 3) compared with the vehicle control group (*P* < 0.01). In each positive control group, the number of metaphases with structural aberrations in the vehicle and positive control groups was within the range established in historical data of the Korea Institute of Toxicology (KIT, 2009). These findings confirm that the methodologies used in this study were valid. Therefore, under the conditions of this test, ORSE showed a positive response in the chromosomal aberration test.

**Table 3:** Chromosomal aberration assay and relative cell counts of ORSE

Nominal conc. of ORSE (μg/mL)	S9 mix	Time hours a)	Mean total aberrant metaphases	Mean total aberrations	Mean of PP + ER	Relative cell counts (%)
6 h treatment (+S9)						
0	+	6–18	2.0/1.0	2.0/1.0	1.0 + 0.0	100
1250	+	6–18	0.0/0.0	0.0/0.0	1.0 + 0.5	108
2500	+	6–18	0.5/0.5	0.5/0.5	0.0 + 0.0	107
5000	+	6–18	1.5/1.5	1.5/1.5	0.0+ 0.0	103
CPA 6	+	6–18	25.5/25.5**	36.0/36.0	0.5 + 0.0	78
6 h treatment (-S9)						
0	-	6–18	0.0/0.0	0.0/0.0	0.5 + 0.0	100
1250	-	6–18	0.0/0.0	0.0/0.0	0.0 + 0.0	103
2500	-	6–18	1.0/1.0	1.0/1.0	0.0 + 0.0	103
5000	-	6–18	0.5/0.5	0.5/0.5	1.5 + 0.0	93
EMS 800	-	6–18	28.5/28.5**	36.0/36.0	0.0 + 0.0	64
22 h treatment (-S9)						
0	-	22-2	0.0/0.0	0.0/0.0	1.0 + 0.0	100
1250	-	22-2	0.5/0.5	1.0/1.0	0.0 + 0.0	103
2500	-	22-2	0.0/0.0	0.0/0.0	0.0 + 0.0	102
5000	-	22-2	0.0/0.0	0.0/0.0	0.0 + 0.0	70
EMS 600	-	22-2	33.0/33.0**	46.5/46.5	0.5 + 0.0	58

**Significantly different from the control at P<0.01

a) Treatment time-recovery time

### MN test

As shown Table 4, there was no significant change in body weight compared with the vehicle control group between the first and final administration of ORSE at 500, 1000, or 2000 mg/kg. The number of MNPCEs/2000 PCEs and PCE/(PCE + NCE) did not increase significantly in the groups treated with SCRT at 500, 1000, or 2000 mg/kg (Table 5). There was a significant increase in the number of MNPCEs/2000 PCEs (50.00) and a significant decrease in the number of PCE/(PCE + NCE) in the positive control group (cyclophosphamide monohydrate), indicating that the present study was performed under good laboratory conditions.

**Table 4:** Body weight changes of Micronucleus test in mice following administration of ORSE

Group	Vehicle control	ORSE	ORSE	ORSE	Positive control (cyclophosphamide monohydrate)
Dose (mg/kg)	0	500	1000	2000	70
Day 1 (Mean ± S.D)	35.9 ± 1.31	37.3 ± 2.34	36.9 ± 1.31	37.2 ± 2.41	37.0 ± 1.93a)
Day 2 (Mean ± S.D)	36.0 ± 1.91	37.3 ± 2.23	36.9 ± 1.48	37.1 ± 2.44	37.7 ± 2.26
Day 3 (Mean ± S.D)	35.5 ± 1.80	37.3 ± 2.46	37.3 ± 1.51	37.4 ± 2.52	37.4 ± 2.64

**Table 5:** Micronucleus test in mice following a single oral dose of ORS

Group	Vehicle control	ORSE	ORSE	ORSE	Positive control
Dose (mg/kg)	0	500	1000	2000	70
MNPCE/2000PCEs (Mean ± S.D)	0.67 ± 0.58	0.33 ± 0.58	2.00 ± 1.00	1.33 ± 0.58	50.00^a^ ± 13.89
PCE/(PCE + NCE) (Mean ± S.D)	0.60 ± 0.06	0.63 ± 0.04	0.58 ± 0.07	0.55 ± 0.12	0.49^a^ ± 0.03
Number of animals	3	3	3	3	3

*MNPCE*: PCE with one or more micronuclei; *PCE*: Polychromatic erythrocyte; *NCE*: Normochromatic erythrocyte

^a^Significantly different from the control at *p* < 0.05

## Discussion

The present study demonstrated that ORSE, a traditional herbal medicine for the treatment of various renal diseases, was not genotoxic using an *in vitro* chromosomal abbreviation test, an *in vitro* bacterial reverse mutation assay, or an *in vivo* MN test. ORSE did not exhibit any genotoxic potential in any of the test systems employed. An *in vitro* chromosome aberration test using CHL cells was performed to determine whether ORSE affects the genotoxicity. The *in vitro* chromosome aberration test is used to identify agents that induce structural chromosomal aberrations in cultured mammalian cells [17]. In the present *in vitro* chromosome aberration test, with 6 h or 22 h treatments regardless of presence or absence of the S9 mix, ORSE did not increase the number of aberrant cells observed. However, to confirm these results, more reliable tests are needed.

*S. typhimurium* strains TA100, TA1535, TA98, and TA1537 and the tryptophan auxotroph strain *E. coli* WP2 *uvr*A were used in the bacterial reverse mutation test [18, 19]. These strains have been shown to be sensitive to the mutagenic activity of a wide range of chemical classes [20].

In the bacterial reverse mutation test (Ames test), we used the histidine auxotroph *S. typhimurium* strains TA100, TA1535, TA98, and TA1537 and the tryptophan auxotroph strain *E. coli* WP2 *uvr*A [18, 19]. In the present study, no mutagenic effects of ORSE in *S. typhimurium* strains TA100, TA1535, TA98, or TA1537, or the *E. coli* WP2 *uvr*A was observed at 555.6, 1666.7, and 5000 μg/plate. The present tested strains have been shown to be sensitive to the mutagenic activity of a wide range of chemical classes. Mutation of genes results in a deficient DNA repair system and greatly enhances the sensitivity of these strains to certain mutagens [21]. Therefore, ORSE did not appear to mutate any genes *in vitro*.

MN tests have been employed for genotoxicity and mutagenicity detection of materials that induce the formation of DNA fragments [22–24]. The increased MN frequency is related to cancer, because MN can be a target of carcinogenesis [25, 26]. In the present study, there was no significant or dose-related increase in the number of MNPCEs per 2000 PCEs at any ORSE-treatment dose level. An elevated frequency of micronucleated PCEs(MNPCEs) indicates chromosomal damage (Fench, Krishna). Also, no abnormal signs and body weights were observed in any of the gropus. Therefore, ORSE is associated with a low risk for carcinogenesis.

## Conclusion

On the basis of our results, the ORSE did not exert any genotoxicity in the battery of three assessments, *in vitro* bacterial reverse mutation assay, or an *in vivo* MN test. To our knowledge, this is the first published study to demonstrated traditional herbal medicine genotoxicity of the ORSE.
